# Academician Qi-Yi Xing (Chi-Yi Hsing): pioneer organic chemist of synthetic insulin

**DOI:** 10.1007/s13238-021-00891-2

**Published:** 2021-11-26

**Authors:** Ming-Hua Qiu

**Affiliations:** State Key Laboratory of Phytochemistry and Plant Resources in West China, Kunming Institute of Botany, Chinese Academy of Science, Kunming, 650201 China

**Figure 1:**
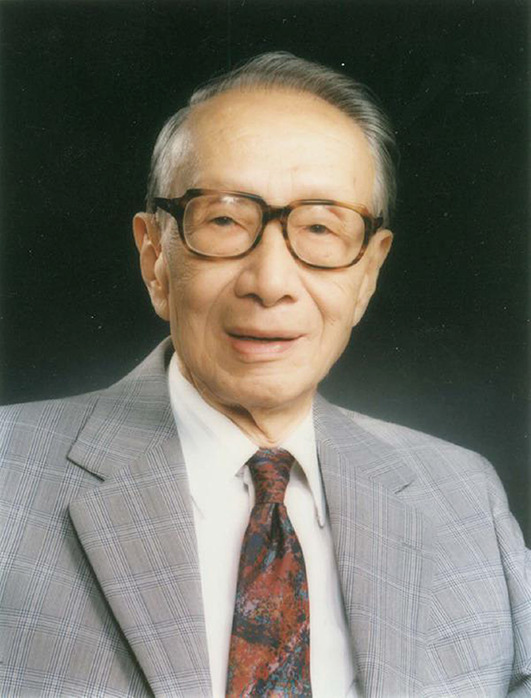
Academician Qi-Yi Xing (Chi-Yi Hsing, November 24th, 1911–November 4th, 2002)

Qi-Yi Xing (Chi-Yi Hsing, November 24th, 1911–November 4th, 2002) (Fig. 1) was one of the remarkable scientist and educators in China. He was a pioneer for Chinese Organic Chemistry development, an Academician of the Chinese Academy of Sciences, and a professor at the College of Chemistry and Molecular Engineering in Peking University. He was one of the leaders of the synthetic crystalline bovine insulin project in China and a person of outstanding talent in the teaching of organic chemistry. On the occasion of 110th anniversary of the establishment of the chemistry department at Peking University, it is also Professor Xing’s 110th birthday, and we pay tribute to his lifelong commitment to the development of organic chemistry, especially protein synthesis, and his teaching style.

QI-Yi Xing was born in November 24th, 1911 in Tianjin, China. His father was a member of the Imperial Academy at the end of Qing dynasty and valued education very highly, which led to him receiving a good education since childhood. In 1933, after graduating from the Chemistry Department of Fu Jen Catholic University at Beijing, he attended University of Illinois at Urbana-Champaign, to pursue a PhD under the supervision of Professor Roger Adams, a world-renowned organic chemist. During the PhD program, he studied the stereochemistry of biphenyl, in which A series of 2,2′-dimethoxy-6,6′-dicaroxybiphenyl were prepared and relative rates of the racemization of various amide substituted compounds were studied in detail (Hsing, 1936). In 1936, he moved to Munich University for his postdoctoral research, where he studied with Professor Heinrich Otto Wieland, a Nobel Prize winner in Chemistry. During the postdoctoral program, he studied toad toxins and completed the structure determination and synthesis of gramine (Xing, 1999). Later, this research discovery became an important method for indole-methylation.

Qi-Yi Xing always clung to the belief that science could save the nation. He left Munich and returned to the Shanghai to work at the Institute of Chemistry in 1937, Academia Sinica as Associate Professor. He started researching Stephania Alkaloids and isolated a new alkaloid which was named Fangchinolin (Chuang et al., 1939), and completed its chemical structure determination (Hsing, 1939). He studied also the separation of unsaturated fatty acids from Castor oil and proposed a simple and practical new method for the determination of unsaturated fatty acid (Hsing, 1939). This method was widely used later.

**Figure 2:**
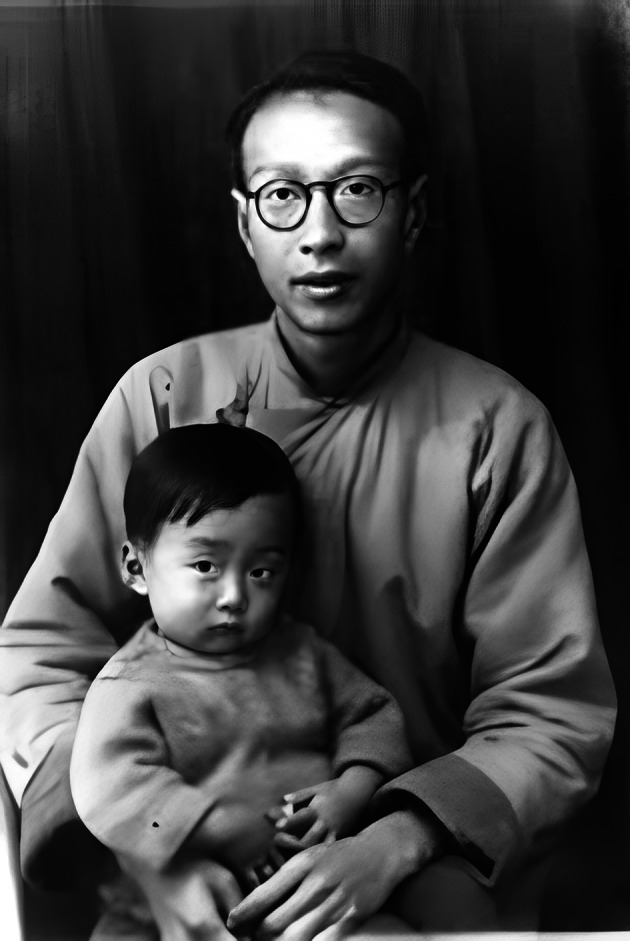
Professor Qi-Yi Xing (Chi-Yi Hsing) of Central China University of Military Medical (1944)

After the Japanese occupied Shanghai, the Institute of Chemistry moved to Kunming. Qi-Yi Xing continued to carry out alkaloid research. In order to cure malaria and support the Chinese Army, he went to the Hekou County on the border with Vietnam to investigate cinchona plant resources and brought back the bark for the separation of quinine (Hsing, 1942). In 1944, Xing served as a Professor in the *Central China University of Military Medical* which affiliated to the New Fourth Army and trained a number of pharmaceutical professional and technical personnel (Qian, 2011) (Fig. 2). Qi-Yi Xing was perhaps the only academician of the Chinese Academy of Sciences to have served in the New Fourth Army. In 1946, he served as Professor in the Department of Agrochemistry and Department of Chemistry at Peking University, he was also Chairman of Chemistry Department of Fu Jen Catholic University at Beijing and was a research Professor in the Institute of Chemistry, Academia Sinica. Throughout the war years, Qi-Yi Xing was still kept on the forefront of organic synthesis research. By studying the action of Grignard Reagents on ethyl ethylenetetracarboxylate, he discovered a general method for the preparation of monoalkylated and monoarylated succinic acids (Hsing, 1949). After the People’s Republic of China was founded, Qi-Yi Xing served as a professor in the Department of Chemistry at Peking University, and remained there until he passed away in November 2002. In 1980, he was elected as an Academician of the Chinese Academy of Sciences in recognition of his distinguished and continuing achievements in original research (Qian, 2011)

In the 1950s, chloramphenicol was the only antibiotic that could be chemically synthesized on a large scale. By studying the stereochemical process of PRINS reaction, Qi-Yi Xing and Gan-Huan Dai et al. designed a new method to synthesize chloramphenicol from the readily available industrial raw material styrene (Hsing, 1957a, b). This method not only theoretically solved the problem of chloramphenicol stereochemistry but was also suitable for large-scale production. The whole synthesis process is only five steps and all of the intermediate products are liquid, which provides favorable conditions for the pipeline transportation and continuous production in the production process (Hsing, 1957a, b). Unfortunately, for various reasons, this new synthesis was not first industrialized in China. In 1968, Carlo Eba Company in Italy used exactly the same method to build a chloramphenicol production line with an annual output of 400 tons. However, the discovery of a new synthetic method of chloramphenicol, pioneered by QI-Yi Xing and his colleagues, received a National Science and Technology Conference Award in 1978.

**Figure 3:**
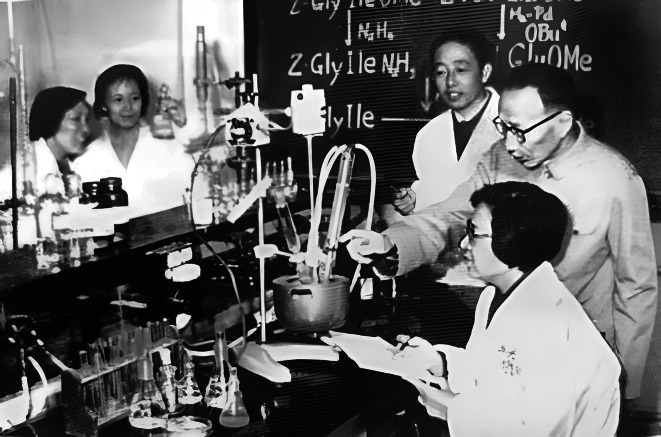
Professor Qi-Yi Xing worked with his students on the experiments of synthesis insulin (1963)

In the early 1950s, protein synthesis was a mysterious and tantalizing field, and many of the world’s top organic chemists were focusing on it. In 1955, British biochemist Dr. Sanger determined the amino acid sequences (primary structure) of the bioactive protein molecule, bovine insulin, by biodegradation and labeling methods for the first time. In 1958, Qi-Yi Xing studied the peptide binding method and terminal group protection of cysteine-containing fragments, also began the synthesis of cysteine-containing polypeptides which laid the foundation for the artificial synthesis of protein insulin (Li, 1963; Loh, 1965) (Fig. 3). In 1959, under the leadership of the National Commission of Science and Technology of China, a big research team brought together in the field of synthetic bovine insulin, by the Shanghai Institute of Organic Chemistry of CAS, the Department of Chemistry of Peking University and Shanghai Institute of Biochemistry of CAS. Qi-Yi Xing was one of the main research leaders of this project. In 1965, Chinese scientists obtained the bioactive protein, crystalline bovine insulin, which was similar to the natural hormone in biological activity and crystalline sharp by artificial synthesis method (Niu, 1964; Wang, 1966). This result immediately attracted the great attention of scientists all over the world, which indicated that Chinese scientists had become leaders in the field of protein and peptide synthesis in the globe. This achievement was awarded the first prize of National Natural Science by the China Government in 1982. The Achievement of Artificial Total Synthetic Bovine Insulin won the “Qiushi Outstanding Scientific and Technological Achievement Group Award” in 1997. Qi-Yi Xing was the leader of this research group.

Traditional Chinese Medicine (TCM) is one of China’s greatest treasures. Qi-Yi Xing devoted great energy to the study on the effective ingredients of TCM. He also noted that most of the TCMs are decocted in water, so repeatedly proposed to carry out the research on the water-soluble components of TCMs, which was a very difficult research project. Ginseng is one of the most famous traditional Chinese medicine in China, many research results of its chemical constituents have been reported, but these often overlook the water-soluble substances such as peptides. Xing’s Group successfully isolated and identified several water-soluble peptides from the famous Ginseng, including γ-GABA and some polypeptides containing cysteine, and further studied their chemical structures and biological activities (Yang, 1990; Long, 1992; Chen, 1996). He opened up a new perspective of thinking and approaching the study of TCM. The achievement won the first prize of Science and Technology Progress by the National Education Commission in 1995.

**Figure 4:**
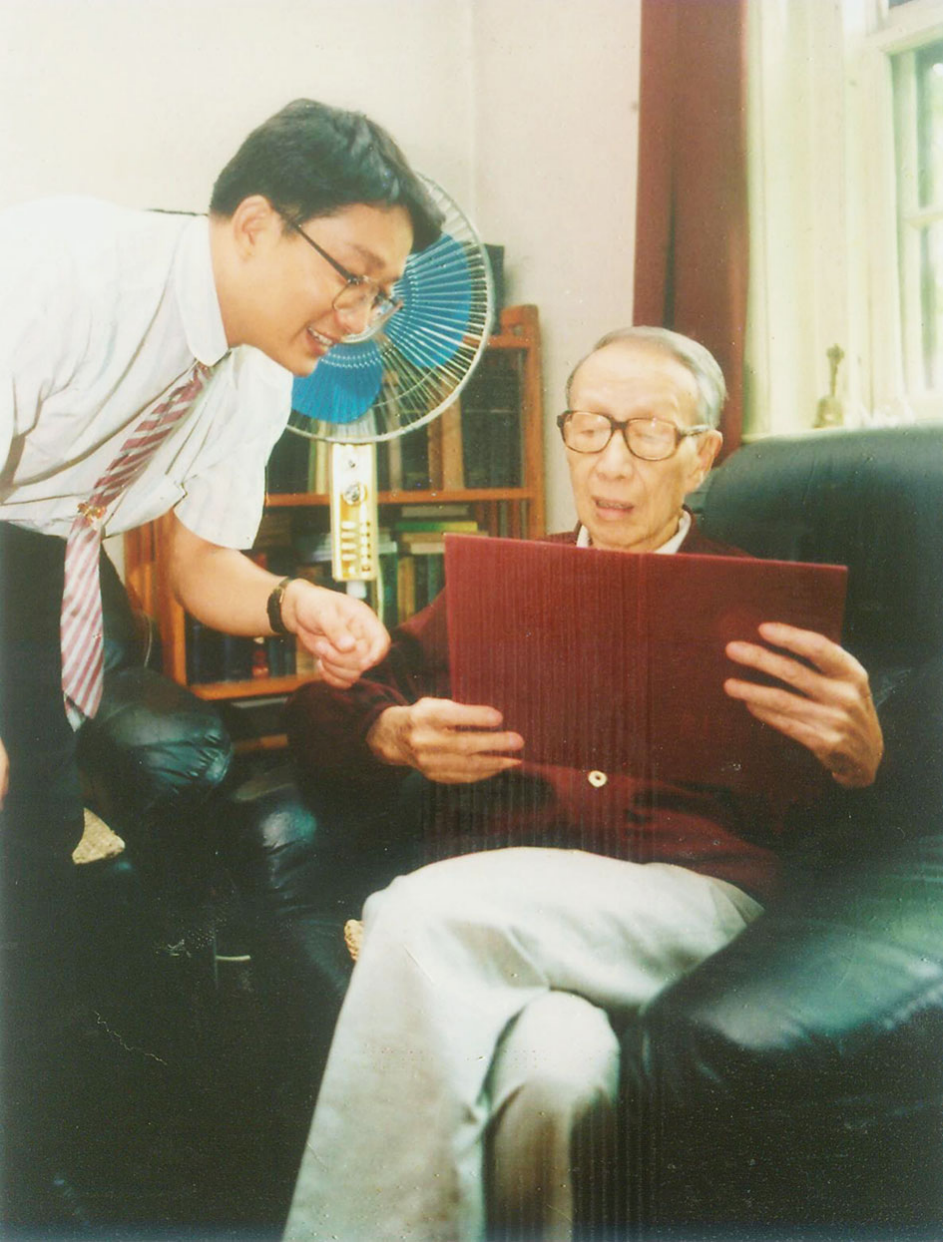
Academician Qi-Yi Xing always tirelessly led his students to think for themselves (1996)

Qi-Yi Xing’s motto in the teaching of organic chemistry was “Hardwork stimulates ideas, Comfort leads to confusion ”(劳则思, 逸则罔). One of the most valuable aspect of his lectures was consistently inspiring students for new ideas. He was a very engaging, compelling and immensely knowledgeable lecturer. He aimed not only at improving his students’ knowledge but also at stimulating their critical and innovative thinking (Fig. 4). He put a lot of effort on the compilation of organic chemistry textbooks. In 1957, Qi-Yi Xing compiled ***Organic Chemistry***, which was the first organic chemistry textbook over millions of words in China. Due to its heavy content, the concise course ***Fundamentals of Organic Chemistry*** was published, which was affectionately known as “Xing’s big textbook”(邢大本) and “Xing’s little textbook”(邢小本). In the 1970s, he led his team to supplement the research results of organic chemistry in the latest 20 years and re-wrote and republished ***Basic Organic Chemistry***. In 1988, the textbook was rated as **Excellent Textbook** by the National Education Commission of China. ***Basic Organic Chemistry*** is both a set of textbooks for higher education and a set of large-scale reference books for all organic chemists, influencing several generations of chemical professional and technicians in China. It was the main reference book for the entrance examination of organic chemistry for graduate students in many universities and research institutions. Qi-Yi Xing’s educational ideas and teaching materials have undoubtedly showed a far-reaching and enormous influence on the discipline of “organic chemistry” in China.

In China, courses on total synthesis of artificial bioactive proteins and organic chemistry cannot avoid mentioning Professor Qi-Yi Xing from Peking University, so indispensable and important were his contributions. On the occasion of 110th anniversary of Qi-Yi Xing’s brith, everyone who has used “Xing’s big textbook”(邢大本) and “Xing’s little textbook”(邢小本) will deeply cherish the memory and commemorate him.
